# Analysis of community older adult care facility construction and demand differentiation based on residential area and population attribute differences

**DOI:** 10.3389/fpubh.2025.1495608

**Published:** 2025-02-12

**Authors:** Yanzhe Liu, Yaolei Wang, Guiping Guo, Tubao Yang, Yupeng Liu, Hongmei Gao, Tiejian Jiang, Xiangmin Li

**Affiliations:** ^1^Xiangya Hospital of Central South University, Changsha, China; ^2^Xiangya School of Public Health, Central South University, Changsha, China; ^3^Hunan Key Laboratory of Epidemiology, Changsha, China

**Keywords:** residential area, crowd attribute, olderly people, older adult care facilities, differentiation

## Abstract

Currently, China has fully entered an aging society. The construction and efficient utilization of community older adult care facilities have become urgent issues that need to be addressed. To explore the differences in the needs of older adult people in the community for older adult care services, this study selected three basic needs: life care, medical security, and cultural and entertainment, as the primary indicators of community older adult care needs. A community older adult care demand indicator system was constructed, which included three primary indicators and 11 secondary indicators. Based on the indicator system, a survey questionnaire was designed, and validity analysis was conducted using Kaiser Meyer Olkin test and Bartlett sphericity test. 490 survey questionnaires were distributed to 22 communities in Wuhan, and 447 valid questionnaires were collected, with a response rate of 91.22%. The results indicated that there were significant differences in the distribution of older adult population in different communities. Different types of residential areas and housing prices affected its construction and use. The demand for home-based older adult care services in communities varied significantly. Through analysis of variance, the type of residential area did not show significant differences in older adult bathing assistance, daytime care, mental comfort, sports activities, etc. (*p* > 0.05), while there were statistical differences in nighttime care, medical care, rehabilitation care, older adult canteens, chess and card entertainment, artistic activities, etc. (*p* < 0.05). No significant difference existed in different housing prices for daytime care, nighttime care, mental comfort, and fitness activities (*p* > 0.05), but there was statistical difference for rehabilitation care, medical care, chess and card entertainment, senior dining halls, and artistic activities (*p* < 0.05). According to logistic regression analysis, the income level, self-care ability, and education level had obvious impacts on their basic needs for life care, with OR values of 11.68, 2.621, and 1.792, respectively. This study can provide effective reference for building diversified community home-based older adult care service facilities.

## 1 Introduction

The aging population worldwide is becoming increasingly prominent. Improving Older Adult Care Facilities (ECF) to satisfy the growing demand for older adult care services has become an important topic in the academic community. The population ageing can be defined as the dynamic process where the number of young people in a given population declines while the number of older adult individuals increases. This demographic shift is primarily driven by a reduction in fertility rates and an extension of life expectancy, which together result in a proportional increase in the older adult population (1, 2). The impact of population aging on economic and social development is multifaceted, including slower economic growth, reduced labor force, increased expenditure on pension insurance, prominent medical issues, and increased social burden (3). Maestas et al. proposed that population aging slowed down economic growth in the United States. The effect of population ageing on per capita GDP growth was estimated based on the demographic profile of each state. The findings revealed a statistically significant negative correlation between the proportion of the population aged 60 and above and per capita GDP, with a 5.5% decline observed for every 10% increase in this demographic. These findings can provide a practical basis for analyzing the impact of current population aging on economic and social development (4). Tu et al. stated that China’s population structure is changing every year with social changes. From 2010 to 2020, the population aged 60 and 65 and over in the Chinese Mainland increased by 5.44 and 4.6%, respectively. Therefore, to deal with changes in population structure, relevant units should improve the care services through new laws and strategies (5). Cardiovascular risk is closely related to the older adult. Moras et al. comprehensively summarized the research and evidence related to the different drugs in the older adult to treat coronary artery disease and acute coronary syndrome in view of the increase in the incidence rate of older adult diseases caused by the aging population. Personalized and comprehensive drug treatment methods for the older adult population were proposed (6). Chen et al. stated that population aging has brought significant social challenges and placed a huge burden on the healthcare system. Therefore, the application of digital technology in the medical field was discussed. The current situation and future development of using digital technology for effective older adult care were summarized, which promoted the development of healthy aging (7). Mason et al. stated that population aging was a global issue. Population changes had profound impacts on countries, regions, and the global economy. In the next few decades, the growth rate of global gross domestic product may decrease by about 1% annually, and high expenditures on children and the older adult may squeeze the living standards of adults (8). Bao et al. said that China is now moving toward an old-age society, and reviewed the current situation of China’s older adult population. China’s population aging began to accelerate in the late 1970s, and continued to grow at an annual rate of about 3.2%. Therefore, improving the quality of life for the older adult is crucial (9).

Older adult care services refer to providing necessary living services for the older adult, meeting their basic material and spiritual needs, and helping to meet their diverse needs. It is an important measure to address population aging (10). The construction of ECF is an important means of enhancing the quality of life (11). Z. Liu et al. established a new spatiotemporal analysis framework to analyze the spatial distribution of ECF from a dynamic perspective, focusing mostly on static features and neglecting the evolution process. The results indicated that older adult stations had significant cluster development characteristics, and the construction of ECF between urban centers and surrounding areas was not balanced (12). Ma et al. stated that home health care is an effective method to solve health problems. A scheduling and routing problem for home health care that considered multiple care centers was proposed. A multi-objective optimization model was constructed to minimize service and latency costs. The model had good performance in solving the scheduling and routing problems (13). Mohd Rosnu et al. conducted an extensive review of relevant literature in data sources such as Web of Science to explore the promotion and barriers to older adult access to healthcare and older adult care services in Southeast Asian countries. The results indicated that accessibility and acceptability were the main influencing factors for older adult people to access medical and older adult care. Therefore, these two factors should be considered when planning medical and older adult care services (14). Geng et al. analyzed the development status of older adult friendly medical institutions abroad to improve older adult care services in China and construct ECF. Suggestions were put forward for older adult friendly medical institutions, like understanding actual needs, increasing human and financial investment, and promoting the construction system from bottom to top (15). Huang et al. quantified and visualized the spatial distribution of community ECF in Shanghai, China based on open source data and geographic information systems. Through global auto-correlation analysis, the accessibility of ECF had spatial clustering characteristics, which provided some reference for older adult people in urban areas to obtain nursing services (16). Given that the deployment plan of robots for internal assistance to the older adult has not yet been practically applied in real life, Bardaro et al. reviewed the application of robots in older adult care services. A new collaborative design toolkit was proposed to link robot capabilities with known older adult factors (17). Mingyu et al. explored the development status, technology, and future prospects of intelligent older adult care digital platforms by considering the application of computer intelligence technology to address the global aging challenge. This provided a certain reference for the development of intelligent older adult care industry (18).

In summary, the population aging has received widespread attention from relevant researchers and extensive research has been conducted on the construction of ECF. However, there are still uneven distribution and low utilization efficiency in current community ECF. Therefore, the study analyzes the layout and construction rationality of community ECF, and explores the optimization method of community ECF through on-site questionnaire surveys. The research aims to provide reliable data support for the layout and construction of ECF in urban communities, thereby optimizing older adult care facility construction in communities. The innovation lies in constructing a community older adult care demand indicator system. Meanwhile, through on-site questionnaire surveys, the actual differences in the needs of different older adult people for community ECF are explored. Through on-site questionnaire surveys, the research can reveal the needs of older adult people for community older adult care facilities, and deeply analyze the problems that exist in the layout and construction process of community older adult care facilities, thereby proposing a more suitable older adult care service model that meets the needs of the older adult. This will help promote the development of community older adult care services and provide scientific basis for relevant policy formulation and planning adjustments.

## 2 Methods

To explore the existing problems in the construction of ECF in the community, this study first analyzed the current layout of ECF in the community using kernel density. Then, the construction rationality of ECF in the community was explored based on accessibility analysis.

### 2.1 Layout analysis of community ECF

**Figure 1:**
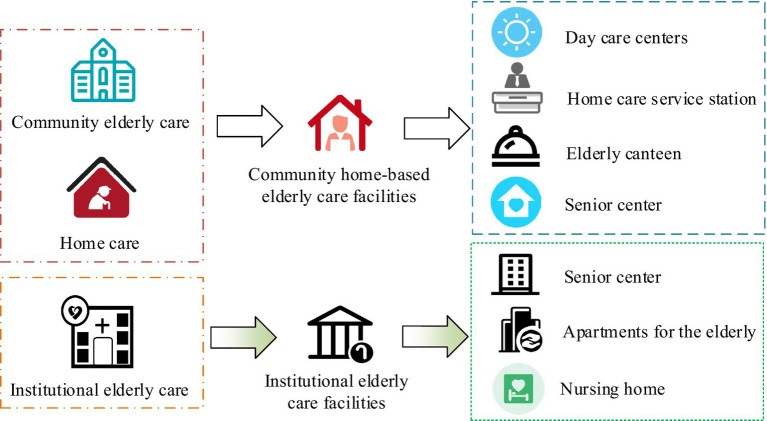
Classification system for ECF.

Older adult care services are usually divided into three types: community care, home-based care, and institutional care. Community older adult care refers to a form of older adult care that is centered around the family, supported by the community, and mainly includes older adult life care, household services, day care, and spiritual comfort (19). Community ECF is one of the main components of ECF, providing basic older adult care services and meeting the spiritual and cultural needs. The classification system of ECF is shown in Figure 1.

A scientifically reasonable layout of ECF in the community can greatly improve the living experience and quality of life, and promote the harmony and health of older adult life. By improving the objective construction of ECF, the subjective needs of the older adult for different older adult care services are considered. At present, old residential areas generally have problems such as inadequate infrastructure and dirty and chaotic community environments, resulting in high demand for aging friendly renovations but few implementations, which affects the quality of life. The resource allocation and utilization efficiency of community ECF is not high, with significant regional and urban–rural disparities in resource allocation. To analyze the layout of ECF in the community, the Nearest Neighbor Index (NNI) was used to determine the spatial distribution type of community ECF and reflect the spatial agglomeration trend of community ECF (20). NNI was calculated based on the average distance between each point feature and its nearest neighbor feature. NNI can well present the spatial distribution characteristics of point features, as shown in Equation 1.


(1) $$N=\overset{N}{\underset{i=1}{\sum }}\frac{\min\left(d_{ij}\right)}{n}/\left(0.5\sqrt{\frac{A}{n}}\right)$$

In Equation 1, $\min\left(d_{ij}\right)$ represents the distance between any point and its nearest neighbor. $\min\left(d_{ij}\right)$ represents the area of the community. n signifies the ECF. A smaller NNI indicates stronger clustering. An increase in value results in a corresponding increase in dispersion. When NNI is equal to 1, the spatial distribution type of community ECF is random. When it is less than 1, it is clustered. When it is greater than 1, it is uniformly distributed (21). To analyze the spatial distribution and density of community ECF, the study used kernel density analysis to measure the strength of older adult care facility clustering (22). Kernel density analysis is a method used to estimate unknown density functions, which solves the distribution density function of a random variable from a given set of sample points. Assuming the density function of the random variable C is $f\left(c\right)$ , the probability density calculation at the community older adult care facility c is shown in Equation 2.


(2) $$f_{n}\left(c\right)=\frac{1}{nh}\overset{n}{\underset{i=1}{\sum }}K\left(\frac{c-c_{i}}{h}\right)$$

**Figure 2:**
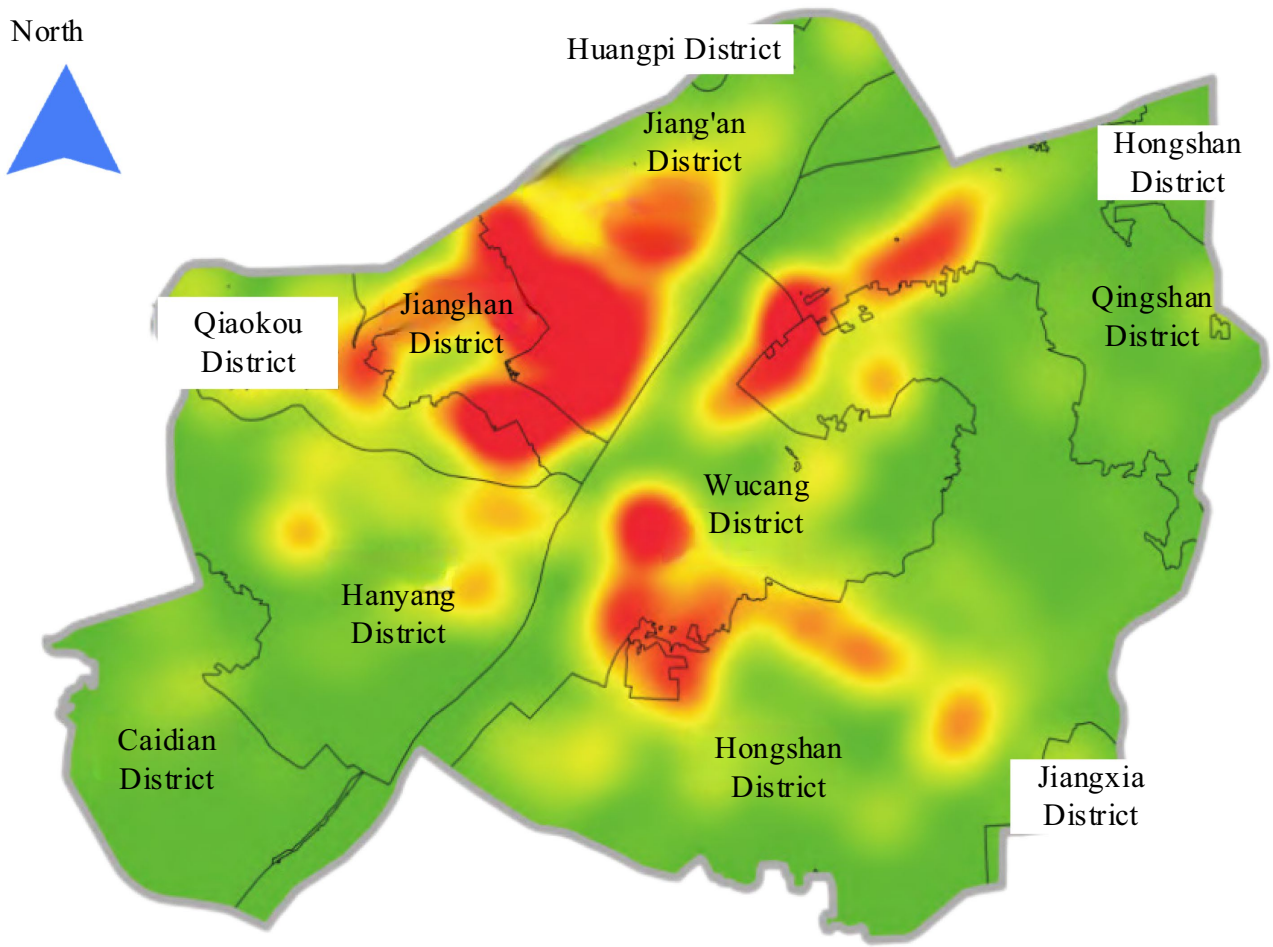
Kernel density of community ECF in Wuhan.

In Equation 2, h represents the radius. $K\left(\frac{c-c_{i}}{h}\right)$ represents the kernel function. $c_{i}$ represents the specific location of ECF. The study takes Wuhan City, Hubei Province, China as an example to explore the spatial layout of community ECF. The kernel density of community ECF is displayed in Figure 2. Red indicates a high kernel density of community ECF, while green indicates a low kernel density.

From Figure 2, there was an uneven distribution in the spatial layout of ECF in Chinese urban communities. Therefore, it is necessary to further analyze the rationality of community ECF construction. According to the actual needs, the community ECF is optimized.

### 2.2 Rationality analysis of community older adult care facility construction

**Figure 3:**
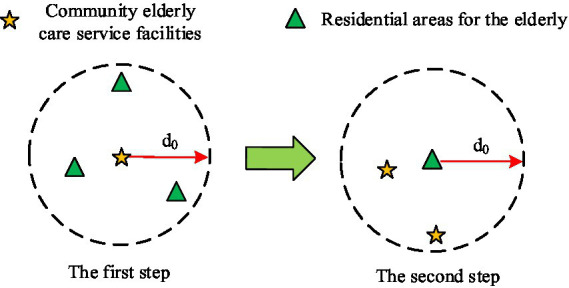
Schematic diagram of TSMS.

From the spatial layout of ECF in Wuhan’s community, there are main problems with uneven distribution and low utilization rate in the current layout of urban community ECF, which leads to the imbalance between supply and demand. Therefore, after analyzing the existing layout of ECF in the community, further analysis should be conducted on the construction rationality of ECF. Accessibility analysis is a collaborative application of geographic spatial data analysis technology and urban transportation system optimization technology. It is one of the crucial indicators for evaluating the layout rationality of public facilities (23). The main purpose of accessibility analysis is to determine the accessibility level of a location or region, providing more comprehensive, accurate, and reliable data support to optimize resource allocation in the decision-making process (24). On the basis of the results of accessibility analysis, it can be determined which areas need to improve the construction of community ECF to meet the needs of older adult care. The study used a Two-Step Mobile Search Method (TSMS) to analyze the spatial accessibility of ECF in the community. TSMS is an important approach for evaluating the accessibility of public service facilities, which calculates the accessibility of service facilities for each demand point through two steps (25, 26). The schematic diagram of the TSMS is displayed in Figure 3.

Firstly, a search area was established, with the community home-based older adult care service facility j as the center, and the straight-line distance from the community where the older adult reside to the community home-based older adult care service facility as the radius $d_{0}$ , to search for all demand points k . Then, the older adult residential area i was taken as the center to search for all facility points j . The supply–demand ratio R and service accessibility A is displayed in Equation 3.


(3) $$\left\{ \begin{array}{c} R=\frac{S_{j}}{\sum_{k\in \left\{d_{kj}\leq d_{0}\right\}}D_{k}}\\ A=\underset{j\in \left\{d_{ij}\leq d_{0}\right\}}{\sum }R \end{array}\right.$$

In Equation 3, $S_{j}$ represents the supply quantity. $D_{k}$ represents the demand quantity. However, the TSMS requires two searches based on supply and demand points, which leads to the demand and supply inflation. The calculated results of demand and supply may be higher than the actual situation, thereby affecting the accuracy of the search results. Therefore, to redistribute demand and supply, weight coefficients were introduced in the study, as shown in Equation 4.


(4) $$\left\{ \begin{array}{c} W_{ij}^{i}=\frac{W_{ij}}{\sum_{i=1}^{n}W_{ij}}\\ W_{ij}^{j}=\frac{W_{ij}}{\sum_{j=1}^{J}W_{ij}} \end{array}\right.$$

In Equation 4, $W_{ij}^{i}$ and $W_{ij}^{j}$ represent the weights from j to i and from i to j , respectively. Gaussian functions are often used to represent how the influence of a phenomenon or service decreases with increasing distance. To better match the travel intention characteristics of the older adult and effectively simulate and describe the law that services gradually weaken with increasing distance, a Gaussian function was used to establish a distance attenuation rule, as shown in Equation 5.


(5) $$W_{ij}=\frac{e^{-\frac{1}{2}{\left(\frac{d_{ij}}{d_{0}}\right)}^{2}}-e^{-\frac{1}{2}}}{1-e^{-\frac{1}{2}}},d_{ij}<d_{0}$$

Based on the weights assigned by the Gaussian equation, the potential impact of supply on demand was calculated to evaluate spatial accessibility. The accessibility of facilities calculated by improved TSMS can better represent the actual allocation level of ECF in the community, reflecting the equality differences in effective allocation of ECF.

### 2.3 Construction of demand indicators

**Figure 4:**
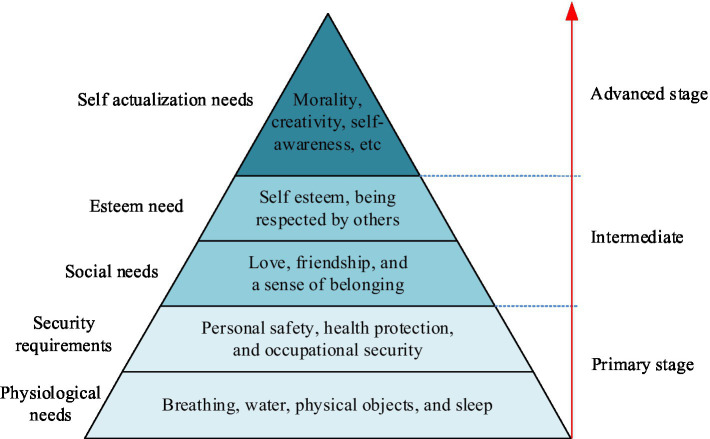
The hierarchical relationship of Maslow’s Hierarchy of Needs Theory.

Exploring the actual needs of older adult people for community ECF can provide reference for the construction of community ECF. Previous researchers typically classified the older adult needs for older adult care services based on their self-care, hierarchy of needs theory, and service nature, to obtain their functional and distance requirements for community ECF. Among them, older adult people can be divided into self-care older adult, self-help older adult, and caregiver older adult according to their degree of self-care (27). Self-care older adult refer to those who are able to fully take care of themselves in their daily lives and do not rely on others for care. Self-help older adult refer to those who rely on handrails, crutches, wheelchairs, and lifting facilities for daily life assistance, and occasionally need help from others. Caregiver older adult refer to those who are unable to fully take care of themselves in their daily lives and require the care of others. Maslow’s Hierarchy of Needs Theory divides human needs into five levels, from basic to complex, namely physiological needs, safety needs, social needs, esteem needs, and self-actualization needs (28). The hierarchical relationship of Maslow’s Hierarchy of Needs Theory is shown in Figure 4.

According to the nature of the service, the needs of the older adult include daily care, healthcare, entertainment, and cultural needs. Among them, the demand for life care mainly involves the daily life care, including services such as diet, daily living, and personal hygiene. Healthcare needs are one of the most important concerns for the older adult, involving health checkups, disease prevention, chronic disease management, rehabilitation care, and other aspects. The demand for entertainment and culture reflects the spiritual and cultural pursuits of the older adult. With the improvement of material life, the older adult begin to pursue higher spiritual satisfaction after meeting their basic living needs. In summary, based on previous literature, the study selected three basic needs of life care, medical security, and cultural and entertainment as the primary indicators of community older adult care demand. A community older adult care demand indicator system consisting of three primary indicators and 11 secondary indicators was constructed, as shown in Table 1.

**Table 1:** Indicator system for community older adult care demand.

Objective	First level indicator	Secondary indicators
Obtain the functional needs of older adult people for community older adult care facilities	Life care needs	Older adult bathing assistance
		Daytime care
		Older adult canteen
		Night care
	Medical security needs	Rehabilitation nursing
		Healthcare
		Spiritual consolation
	Cultural and entertainment needs	Chess and card entertainment
		Sports activities
		Art activities
		University for the older adult

From Table 1, the community older adult care demand indicator system constructed in the study covered the physiological needs, emotional needs, safety needs, self-actualization needs, and respect needs of the older adult. A survey questionnaire was designed to obtain the functional and distance requirements of older adult people for community ECF, and provide reference for determining the service content and scope of community ECF. The first part of the survey questionnaire collects basic information about the older adult by investigating their gender, age, level of self-care, income, and type of residential area. The second part is the 11 secondary indicators of community older adult care demand proposed in the study, and use the Likert scale to investigate the degree of demand and walking time requirements. The Likert Scale is a scoring summative scale used to measure an individual’s attitude or opinion toward a specific thing. For the convenience of analyzing and comparing different results, the study used the Likert five point scale, which was divided into five categories: not needed (1), occasionally needed (2), generally needed (3), somewhat needed (4), and very needed (5). To obtain the distance requirements of older adult people for community ECF, the study scored them based on walking time, divided into 5 min or less (1), 5–10 min (2), and 10–15 min (3). To ensure the expert validity of the questionnaire, experts in the field of older adult care were invited to review the questionnaire before formal use. To ensure that the questionnaire design is reasonable, the questions are accurate, and can truly reflect the research objectives. To obtain a representative sample and more accurately reflect the overall situation, the study adopted a sampling survey method to select different types of survey subjects according to a certain proportion, including gender, education level, and economic status. In addition, to ensure a balanced sample distribution, the study conducted sampling in multiple communities to ensure that each community had an appropriate number of samples.

After obtaining the questionnaire data, the study conducted validity and reliability analysis using SPSS 22.0. Validity analysis refers to the measurement accuracy of a scale, mainly used to evaluate whether the measuring tool or method can accurately measure the content or topic to be measured. The study used Kaiser-Meyer-Olkin (KMO) test and Bartlett sphericity test for validity analysis. The KMO test statistic compares the simple correlation coefficient and partial correlation coefficient between variables. It usually falls between 0 and 1. A value that is closer to 1 indicates a stronger correlation between variables (29). Bartlett sphericity test is a statistical method used to test whether the pairwise covariance between variables is equal in multivariate statistical analysis. It is commonly used to evaluate the correlation between original variables. Reliability refers to the consistency in the results obtained by repeatedly measuring the same object using the same method. The study used the Cronbach’s *α* reliability coefficient method for reliability analysis (30, 31). The Cronbach’s α reliability coefficient method is applied to measure the internal consistency of a test. This method calculates the proportion of the sum of covariance between the various items that make up the test in the total variance of the test score to obtain a reliability coefficient α. A larger α value indicates higher reliability. When α is greater than 0.9, it indicates high reliability. As α decreases, the reliability gradually decreases until α is less than 0.6, indicating poor reliability.

## 3 Results

To analyze the differences in older adult care needs among community residents, the study first conducted statistical and reliability analysis on the questionnaire results, and separately analyzed the types of residential areas, housing prices, and demographic attributes within the survey scope. Finally, a feature analysis was conducted on the functional requirements of community ECF, including descriptive analysis, differentiation analysis, and correlation analysis.

### 3.1 The impact of residential area types and housing prices on older adult care demand

A total of 490 survey forms were sent out to people in Wuhan and got back 447 complete ones, with an effective response rate of 91.22%. The basic information of the survey subjects was presented in Table 2. The proportion of people aged 60 to 70 in the valid questionnaire was 48.55%, and people aged 70 and above was 51.45%, which was basically balanced. There were 231 females and 216 males, respectively, with basically balanced gender ratio. The majority of survey respondents had a low level of education, with 52.12% being elementary school or below. The highest proportion of population with income between 3,000 and 5,000 RMB was 60.18%. The lowest proportion of people with an income of over 8,000 RMB was 5.14%.

**Table 2:** Basic information of the survey subjects.

Investigation content	Classification	Number	Proportion/%
Age	60–70	217	48.55
	>70	230	51.45
Gender	Male	216	48.32
	Female	231	51.68
Educational level	Elementary school and below	233	52.12
	Middle school	169	37.81
	University and above	45	10.07
Income level/RMB	<3,000	80	17.90
	3,000–5,000	269	60.18
	5,000-8,000	75	16.78
	>8,000	23	5.14

**Figure 5:**
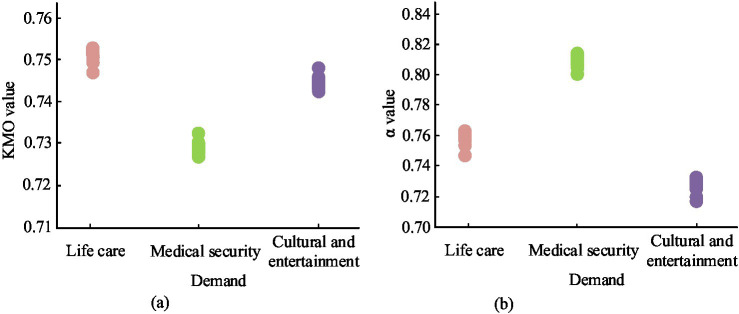
Reliability and validity analysis results. **(A)** Validity analysis. **(B)** Reliability analysis.

After testing, the reliability and validity analysis results of the three primary indicators of life care demand, medical security demand, and cultural and entertainment demand were shown in Figure 5. From Figures 5A,B, the *α* values of the three primary indicators of life care demand, medical security demand, and cultural and entertainment demand were 0.766, 0.820, and 0.747, and the KMO was 0.756, 0.731, and 0.750, proving that the reliability and validity tests were qualified.

Further reliability analysis was conducted on each secondary indicator. The reliability analysis was presented in Table 3. Among the needs for life care, the corrected item-total correlation for night care had the highest overall correlation, at 0.665, demonstrating the highest contribution to the total score. In the demand for medical security, the corrected item-total correlation in healthcare was the highest, at 0.866. In terms of cultural and entertainment needs, the corrected item-total correlation for artistic activities was the highest, at 0.511. The α values were all greater than 0.7, and the highest reached 0.819. The results indicated that the questionnaire survey data collected in this study was stable and reliable.

**Table 3:** Requirement reliability analysis.

First level indicator	Secondary indicators	Corrected item-total correlation	α
Life care needs	Older adult bathing assistance (S1)	0.535	0.765
	Daytime care (S2)	0.634	
	Older adult canteen (S3)	0.446	
	Night care (S4)	0.665	
Medical security needs	Rehabilitation nursing (S5)	0.369	0.819
	Healthcare (S6)	0.866	
	Spiritual consolation (S7)	0.864	
Cultural and entertainment needs	Chess and card entertainment (S8)	0.394	0.745
	Sports activities (S9)	0.464	
	Art activities (S10)	0.511	
	University for the older adult (S11)	0.424	

**Figure 6:**
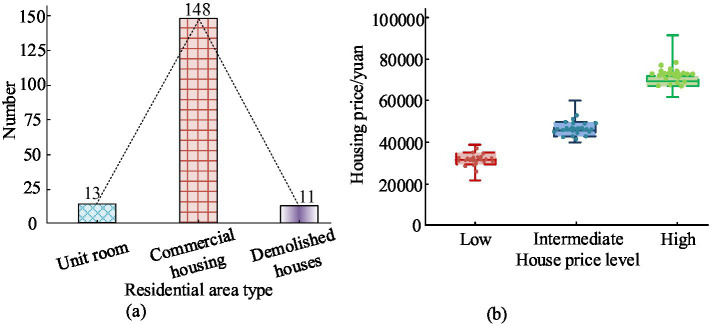
Statistical results of residential area types and housing prices. **(B)** Residential area type. **(B)** Cluster distribution of commodity housing prices.

The survey scope covered a total of 20 communities and 172 residential areas. The statistical results of using K-means clustering to analyze the housing prices, residential area types, and housing prices of commercial housing were shown in Figure 6. From Figure 6A, the survey scope of the study included 13 unit houses, 148 commercial houses, and 11 demolished houses. From Figure 6B, low priced commercial housing stands for commercial housing priced below 39,000 RMB. Medium priced commercial housing stands for commercial housing within 39,000–62,000 RMB. High priced commercial housing refers to commercial housing priced above 62,000 RMB. The types of residential areas within the survey scope were divided into three categories: unit houses, commercial houses, and demolished houses, with the number of commercial houses being significantly higher than that of unit houses and demolished houses.

### 3.2 The influence of population attributes on the older adult care needs

**Figure 7:**
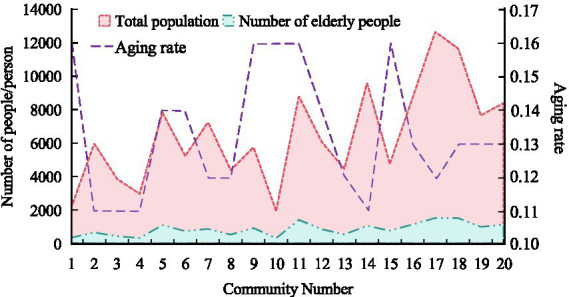
Current situation of population aging.

The current population aging in 20 communities within the survey scope was shown in Figure 7. From Figure 7, there were significant differences in the distribution of older adult population among these 20 communities, with the highest aging rate being 0.16 and the lowest being 0.11. There were certain differences in the aging rate among different communities within the survey scope.

### 3.3 Descriptive analysis

**Figure 8:**
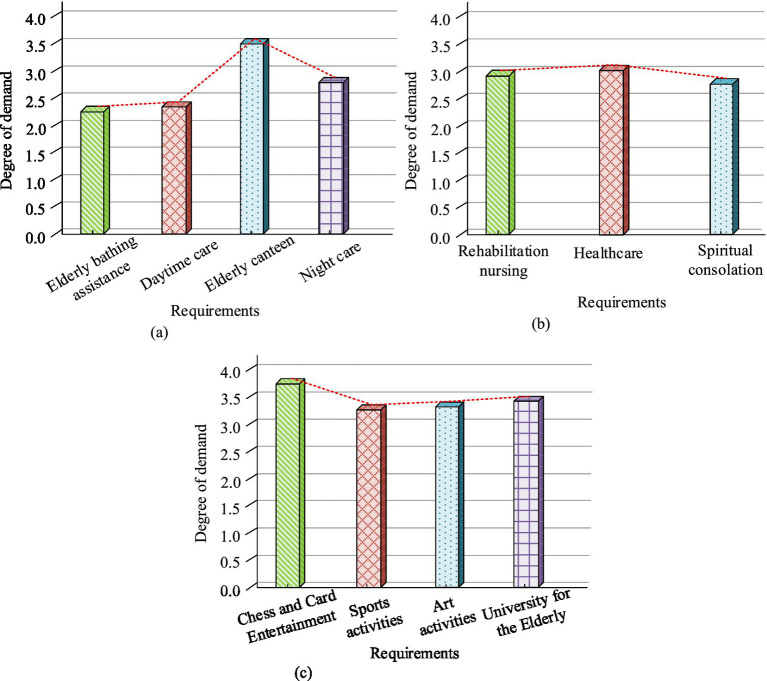
Overall demand for community ECF. **(A)** Life care needs. **(B)** Medical security needs. **(C)** Cultural and entertainment needs.

According to statistics, the overall demand for community ECF among the survey subjects was shown in Figure 8. From Figure 8A, the average demand for life care was 2.72, with the highest demand for older adult canteens at 3.50. The demand for older adult bath assistance was the lowest, at 2.25. From Figure 8B, the average demand for medical security was 2.86, with healthcare having the highest demand at 3.06. The need for spiritual comfort was the lowest, at 2.72. From Figure 8C, the average demand for cultural and entertainment was 3.46, with the highest demand for chess and card entertainment at 3.72. The results showed that the average overall demand for community ECF among the survey respondents was 3.03, which was moderate. The cultural and entertainment needs were higher than living care needs and medical security needs.

**Figure 9:**
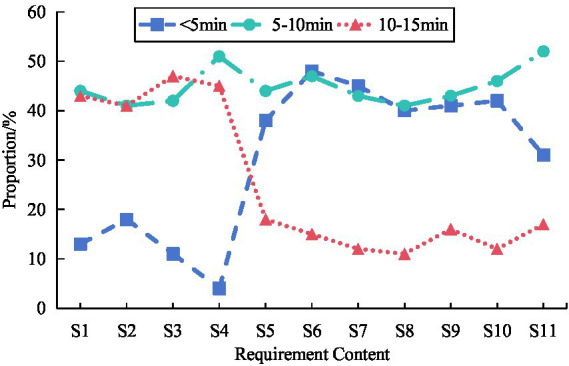
Proportion of survey respondents’ distance requirements for community ECF.

The study evaluated the walking time to determine the spatial accessibility needs of survey participants for different functions of community ECF. The proportion of distance demand for community ECF among survey respondents was shown in Figure 9. The survey respondents had the highest demand for distance to healthcare, with 48.27% of respondents hoping to walk within 5 min to access healthcare services. The survey respondents had the lowest distance requirement for night care, with only 3.98% of them hoping to walk within 5 min to obtain night care services.

### 3.4 Differentiation analysis

**Figure 10:**
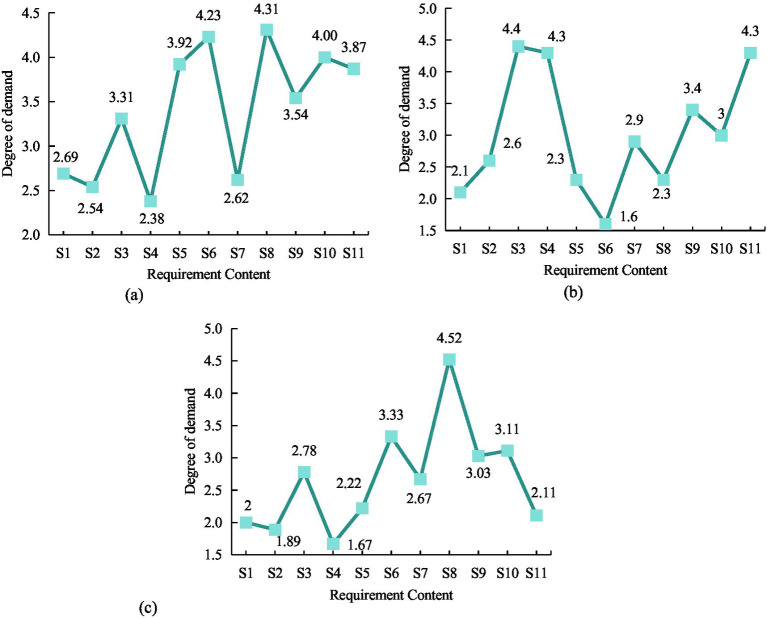
The average demand level of older adult people in different types of residential areas. **(A)** Commercial housing. **(B)** Unit room. **(C)** Demolished houses.

The statistical results of the average demand for community ECF among older adult people of different types of residential areas were shown in Figure 10. From Figure 10A, older adult people living in commercial housing had the highest demand for chess and card entertainment services, at 4.31, and the lowest demand for nighttime care, at 2.38. From Figure 10B, older adult people living in unit rooms had the highest demand for older adult canteen services, at 4.4, and the lowest demand for healthcare, at 1.6. From Figure 10C, the older adult living in the demolished houses had the highest demand for chess and card entertainment services, at 4.52, and the lowest demand for night care, at 1.67. Therefore, there was significant difference in the impact of residential area type on night care, medical care, rehabilitation care, older adult canteens, chess and card entertainment, and artistic activities (*p* < 0.05). The results indicated that different types of residential areas to some extent affected the demand of older adult people for community ECF.

**Figure 11:**
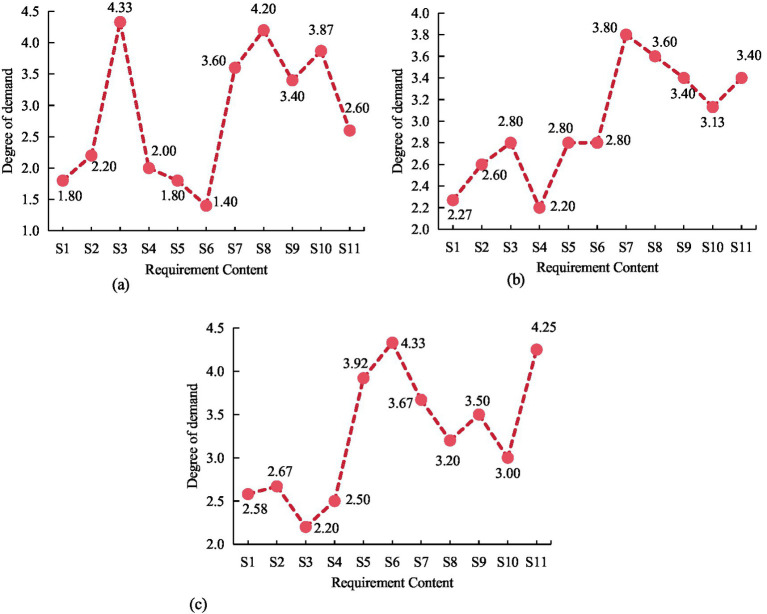
The average demand level of older adult people for different commodity housing prices. **(A)** Low-priced. **(B)** Medium-priced. **(C)** High- priced.

The statistical results of the average demand for community ECF among older adult people with various housing prices were shown in Figure 11. From Figure 11A, older adult people living in low-priced commercial housing had the highest demand for older adult canteen services, at 4.33, and the lowest demand for healthcare services, at 1.40. From Figure 11B, older adult people living in medium-priced commercial housing had the highest demand for mental comfort services, at 3.80, and the lowest demand for nighttime care, at 2.60. From Figure 11C, older adult people living in high-priced commercial housing had the highest demand for healthcare services, at 4.33, and the lowest demand for older adult canteens, at 2.20. Therefore, there was no significant difference in the impact of different housing prices on daytime care, nighttime care, mental comfort, and fitness activities (*p* > 0.05). Significant difference existed in the impact of different housing prices on rehabilitation nursing, healthcare, chess and card entertainment, older adult canteens, and artistic activities (*p* < 0.05). The results indicated that different prices of commercial housing also to some extent affected the demand of older adult people for community ECF.

### 3.5 Correlation analysis

To explore the correlation between population attributes and community older adult care service facilities, logistic regression analysis was used, with self-care ability, income level, and education level as independent variables, and service function demand level as the dependent variable. The correlation analysis results are displayed in Table 4. Self-care ability, income level, and education level had an obvious impact on the needs for life care, medical security, and cultural and entertainment (*p* < 0.05). The OR values of self-care ability, income level, and education level on the demand for life care were 2.621, 11.68, and 1.792, respectively.

**Table 4:** Correlation analysis results.

Demand	Crowd attributes	Error	z	*p*	OR
Life care needs	Self-care ability	0.286	3.357	0.001*	2.621
	Income	0.286	8.565	0.000*	11.680
	Educational level	0.241	2.416	0.016*	1.792
Medical security needs	Self-care ability	0.387	3.644	0.000*	4.129
	Income	0.535	2.860	0.004*	4.599
	Educational level	0.854	3.325	0.001*	17.198
Cultural and entertainment needs	Self-care ability	0.273	3.011	0.003*	1.442
	Income	0.234	7.903	0.000*	6.371
	Educational level	0.212	0.342	0.032*	1.077

*p < 0.05.

## 4 Conclusion

To explore the layout and construction rationality of urban community ECF, a community older adult care demand indicator system was constructed and a questionnaire survey was conducted. The results showed that the average demand for life care among the survey subjects was 2.72, the average demand for medical security was 2.86, and the average demand for cultural and entertainment was 3.46. The survey respondents had the highest demand for distance in healthcare, with 48.27% of them hoping to walk within 5 min to access healthcare services. The older adult living in commercial housing had the highest demand for chess and card entertainment services, at 4.31, and the lowest demand for nighttime care, at 2.38. The older adult living in unit rooms had the highest demand for older adult canteen services, at 4.4, and the lowest demand for medical care, at 1.6. The older adult living in the demolished houses had the highest demand for chess and card entertainment services, at 4.52, and the lowest demand for night care, at 1.67. The older adult living in low-priced commercial housing had the highest demand for older adult canteen services, at 4.33, and the lowest demand for healthcare services, at 1.40. The older adult living in medium priced commercial housing had the highest demand for mental comfort services, at 3.80, and the lowest demand for nighttime care, at 2.60. The older adult living in high priced commercial housing had the highest demand for healthcare services, at 4.33, and the lowest demand for older adult canteens, at 2.20. The self-care ability, income level, and education level had obvious impacts on the needs for life care, medical security, and cultural and entertainment (*p* < 0.05). The research results were similar to those of Zhang and Huang. Zhang et al. pointed out that promoting the upgrading of supply and demand of older adult care service resources can start from the needs of the older adult and increase investment in infrastructure construction and older adult care service resources. Huang et al. indicated that the spatial distribution of community older adult care facilities in China is unbalanced, and accessibility has spatial agglomeration characteristics, leading to low efficiency in resource allocation of older adult care facilities (16). However, these studies did not analyze the older adult retirement needs. The research results are of great significance for promoting the development of older adult care services. Rong et al. also pointed out that home-based older adult care systems play an important role in meeting the older adult care needs of developing countries (32). However, the study only conducts a questionnaire survey on some communities in the city. The survey results may not be comprehensive enough. Therefore, in future research, the research scope should be further expanded to obtain more comprehensive research conclusions, thereby promoting the rationality of urban community older adult care facility construction.

## Data Availability

The original contributions presented in the study are included in the article/supplementary material, further inquiries can be directed to the corresponding authors.
